# Pharmacological and toxicological studies of a novel goserelin acetate extended-release microspheres in rats

**DOI:** 10.3389/fphar.2023.1125255

**Published:** 2023-02-21

**Authors:** Mao Yutong, Ye Liang, Sha Chunjie, Guan Xiaolin, Gong Xiaoyan, Dong Lin, Du Guangying, Zhang Xuemei, Cen Xiaobo, Tian Jingwei, Yu Pengfei, Wang Hongbo

**Affiliations:** ^1^ School of Pharmacy, Key Laboratory of Molecular Pharmacology and Drug Evaluation (Yantai University), Ministry of Education, Collaborative Innovation Center of Advanced Drug Delivery System and Biotech Drugs in Universities of Shandong, Yantai University, Yantai, China; ^2^ School of Pharmacy, Binzhou Medical University, Yantai, China; ^3^ State Key Laboratory of Long-Acting and Targeting Drug Delivery System, Shandong Luye Pharmaceutical Co., Ltd., Yantai, China; ^4^ WestChina-Frontier PharmaTech Co., (WCFP), National Chengdu Center for Safety Evaluation of Drugs (NCCSED), Chengdu, China

**Keywords:** goserelin acetate extended-release microspheres, pharmacology and toxicology study, GnRH agonist, LY01005, acute-on-chronic phenomenon

## Abstract

LY01005 is an investigational new drug product of goserelin acetate which is formulated as extended-release microspheres for intramuscular injection. To support the proposed clinical trials and marketing application of LY01005, pharmacodynamics, pharmacokinetics and toxicity studies were performed in rats. In the pharmacological study in rats, LY01005 induced an initial supra-physiological level increase of testosterone at 24 h post-dosing which then rapidly fell to castration level. The potency of LY01005 was comparable to the comparator Zoladex^®^ but its effect lasted longer and more stable. A single-dose pharmacokinetics study in rats demonstrated that the C_max_ and AUC_last_ of LY01005 increased in a dose-proportional manner in the range of 0.45–1.80 mg/kg and the relative bioavailability was 101.0% between LY01005 and Zoladex^®^. In the toxicity study, almost all of the positive findings of LY01005 in rats including the changes in hormones (follicle-stimulating hormone, luteinizing hormone, testosterone, progestin) and in reproductive system (uterus, ovary, vagina, cervix uteri, mammary gland, testis, epididymis and prostate) were related to the direct pharmacological effects of goserelin. Mild histopathological changes in foreign body removal reaction induced by excipient were also observed. In conclusion, LY01005 displayed a sustained-release profile of goserelin, and exerted a continuous efficacy *in vivo* in animal models, which had a comparable potency but with a more sustained effect than that of Zoladex^®^. The safety profile of LY01005 was largely the same with Zoladex^®^. These results strongly support the planned LY01005 clinical trials.

## 1 Introduction

Goserelin is a potent synthetic decapeptide agonist analogue of the naturally occurring hormone known as gonadotropin releasing hormone (GnRH) (Chrisp and Goa, 1991). The pharmacological effects of goserelin are related to its occupation of the majority of GnRH receptors present on the pituitary which then become internalized, disappearing from the cell surface. As a result of the receptor occupancy, there is an initial surge of follicle-stimulating hormone (FSH) and luteinizing hormone (LH) secretion. Then, the secretions of FSH and LH are markedly suppressed due to the desensitization of GnRH receptors caused by the continued presence of goserelin. In males, this suppression leads to testes atrophy, suppression of testosterone secretion and prostate involution. In females, this will result in ovarian atrophy, a decrease in estradiol to castrate or post-menopausal values and involution of the uterus and mammary gland, as well as regression of sex hormone-responsive tumors (Brogden and Faulds, 1995; Cheer et al., 2005).

As compared to daily drug administration, depot formulations and implantable devices have many advantages. They can enhance patient compliance and reduce the total dose of drug required to achieve castrate testosterone levels. They can also minimize tissue damages related to frequent injections. Zoladex^®^, a long-acting subcutaneous (s.c.) implantable formulation of goserelin acetate, was developed by AstraZeneca UK Limited and approved by FDA. It is indicated for the management of locally confined carcinoma of the prostate and palliative treatment of advanced carcinoma of the prostate in men. It is also indicated for the management of endometriosis and used as an endometrial-thinning agent prior to endometrial ablation for dysfunctional uterine bleeding in women and palliative treatment of breast cancer in pre- and perimenopausal women. However, implant formulations employed by Zoladex^®^ require the concurrent administration of a local anesthetic or a special injection technique (Perren et al., 1986; Filicori et al., 1993; Nukui and Morita, 2011). It is clear that there is clinical need to improve the administration convenience and patient compliance of the drug. This can be achieved by developing an injectable goserelin formulation to avoid the issues related to implant formulation.

Goserelin acetate extended-release microspheres for injection (Code name: LY01005) is a novel liquid intramuscular (i.m.) injection of goserelin acetate developed by Luye Pharmaceutical Co., Ltd. (Luye pharm). Unlike Zoladex^®^ implant (using 16-gauge needle), LY01005 can be given through a fine 21-gauge needle. As such, LY01005 does not require the concurrent administration of a local anesthetic or a special injection technique (e.g., using ice cubes or vapo-coolant spray for relieving pain induced by Zoladex^®^ implant injection). LY01005 could minimize the discomfort to patients and reduce the risk of injection site hematoma (especially important for patients who also take anticoagulants). In addition, LY01005 only requires conventional injection method to deliver the drug which can be done by a physician or other members of the healthcare team. The injection frequency can be tailored to enable more individualized, patient-orientated treatment, which can be given at home or in the office, and timed to coincide with regular check-ups.

Here we report a series of pharmacodynamics, pharmacokinetics, and toxicological studies of LY01005. Using Zoladex^®^ as a comparator, these studies were intended to support the clinical trials and marketing application of LY01005 as a reformulated drug product.

## 2 Materials and methods

### 2.1 Chemicals and reagents

LY01005 was provided by Luye Pharma. The active ingredient, goserelin acetate, was micro-encapsulated in PLGA at a concentration of 40 mg of goserelin acetate per gram of microspheres (4% drug content). The diluent for i. m. injection was a sterile, clear and colorless solution, containing carboxymethylcellulose sodium (SCMC), sodium chloride and water for injection. Placebo microspheres (without goserelin acetate) and vehicle (1% SCMC) were also supplied by Luye Pharma. LY01005 and placebo microspheres were suspended in SCMC to the desired concentrations.

### 2.2 Animals and ethics statement

Adult male and female Sprague-Dawley rats were purchased from Beijing Vital River Laboratory Animal Technology Co., LTD. (Beijing, China). Rats were quarantined and acclimatized to the housing environment for 8 days before the start of the experiments. The procedures related to the animal experiments complied with the relevant laws and regulations in the experimental animal use and management and the relevant requirements of the *Institutional Animal Care and Use Committee* (IACUC) at respective research institutes. The safety pharmacology studies, acute and subchronic toxicity studies were complied with *Good Laboratory Practice Regulations for Non-clinical Research* (GLP) by National Medical Products Administration (NMPA), Organization for Economic Co-operation and Development (OECD) Principles on *Good Laboratory Practice ENV/MC/CHEM (98)17*, and *21 CFR Part 58 of FDA Good Laboratory Practice for non-clinical laboratory studies.*

### 2.3 Pharmacology studies

#### 2.3.1 Single-dosing pharmacology study in rats

Forty-eight male rats were randomly assigned to six groups: control group (38.3 mg/kg placebo microspheres, i. m.), castration group (rats were castrated and treated with 38.3 mg/kg placebo microspheres, i.m.), Zoladex^®^ group (1.44 mg/kg goserelin, s.c.), and LY01005 groups (0.36, 0.72 and 1.44 mg/kg goserelin, i. m.). Blood samples (approximate 200 μL) were collected on Day 0 (prior to dosing) and days 1, 2, 4, 7, 10, 14, 18, 21, 24, 28, 32 and 35 after injection. Blood samples without anticoagulation were centrifuged at 3,000 rpm for 15 min at room temperature. Serum testosterone levels were analyzed using the Testosterone Parameter Assay Kit (R&D Systems, Inc., United States) according to the manufacturer’s instructions.

#### 2.3.2 Multiple-dosing pharmacology study in rats

Thirty-two male rats were randomly assigned to four groups: control group (38.3 mg/kg placebo microspheres, i.m.), castration group (rats were castrated and treated with 38.3 mg/kg placebo microspheres, i.m.), Zoladex^®^ group (1.44 mg/kg goserelin, s.c.), and LY01005 group (1.44 mg/kg goserelin, i.m.), which would receive respective treatments once every 28 days for a total of three doses. Blood samples (approximate 200 μL) were collected on Day 0 (prior to dosing) and days 1, 4, 7, 14, 21, 28, 35, 42, 49, 56, 63, 70, 77 and 84 after injection. Blood samples without anticoagulation were centrifuged at 3,000 rpm for 15 min at room temperature. Serum testosterone levels were analyzed using the Testosterone Parameter Assay Kit (R&D Systems, Inc., United States) according to the manufacturer’s instructions.

#### 2.3.3 Functional observational battery test in rats

Functional observational battery (FOB) tests were performed to evaluate the potential effects of LY01005 on neurobehavioral functions in rats. Rats were randomly assigned to the following groups: vehicle control (1% SCMC), placebo microspheres (287.54 mg/kg) and LY01005 at doses of 1.2, 3.6, 10.8 mg/kg, with 10 rats (n = 5/sex) per group. Animals received a single i.m. injection with the same dose volume of 3 mL/kg. The neurobehavioral performance of animals was assessed with the FOB tests, including home-cage observation, hand-held observation, open-field observation, stimulus response observation, grip strength test and body temperature test, at 1 h, 6 h, Days 2, 7, 11, 14 and 28 postpose.

#### 2.3.4 Effect of LY01005 on the respiratory function in conscious rats

Conscious rats were used to evaluate the potential effect of LY01005 on the respiratory function by DSI Respiratory Whole Body Plethysmography System. The rats were randomly assigned to groups of vehicle control (1% SCMC), placebo microspheres, or LY01005 at doses of 1.2, 3.6 and 10.8 mg/kg, respectively, with a dose volume of 3 mL/kg. The parameters of respiration rate, tidal volume and minute ventilation rate were evaluated at 1, 72, 240 and 648 h after i.m. injection.

### 2.4 Pharmacokinetics study in rats

Male rats were randomly divided into three groups (n = 6/group) and were administered with a single i.m. dose of 0.45, 0.90 or 1.80 mg/kg of LY01005 (peptide base). The comparison experiment between LY01005 and Zoladex^®^ at the dosage of 1.80 mg/kg was performed in male rats (n = 6/group). Blood samples (0.5 mL) were collected from the eye socket prior to dosing and at 0.5, 1, and 6 h after injection on day 1 and on days 2, 4, 7, 9, 10, 11, 13, 15, 18, 21, 24 and 28. The plasma concentrations of goserelin were determined by LC-MS/MS. The pharmacokinetic parameters of goserelin were calculated using non-compartmental methods by the software Phoenix WinNonlin 6.3 (Pharsight, Mountain View, CA, United States).

### 2.5 Toxicology studies

#### 2.5.1 Acute toxicity study in rats

Rats (n = 5/sex/group) were i.m. injected with a single dose of vehicle control (1% SCMC), placebo microspheres, or LY01005 at doses of 3.75, 15 and 60 mg/kg, respectively, using a dose volume of 12 mL/kg. The dosing day was defined as Day 1. Parameters for evaluation included the mortality, clinical signs, body weights, food consumption, hematology, clinical chemistry, and gross- and microscopic-pathology evaluations. Scheduled necropsies were conducted in all animals on Day 29.

#### 2.5.2 Sixteen-week subchronic toxicity study in rats

Rats (n = 15 rat/sex/group) were administered with i.m. injection of vehicle (1% SCMC), placebo microspheres (287.54 mg/kg) or LY01005 at doses of 1.2, 3.6 or 10.8 mg/kg, respectively, once every 4 weeks for 16 weeks followed by an 8-week recovery period. Drug was injected in the long adductor of the hindlimb using a dosing volume of 3.0 mL/kg. At the end of the treatment and recovery periods, 10 rats/sex/group and five rats/sex/group were euthanized for pathological evaluations, respectively. Parameters evaluated included the mortality, clinical signs, body weight, food consumption, hormone levels, hematology, clinical chemistry, organ weights, gross- and microscopic pathology.

The toxicokinetics study was performed in combination with the subchronic toxicity study. Each LY01005 group included 24 rats (n = 12/sex/group) for TK study. Blood samples were collected from the rats in LY01005 groups at pre-dose (0 h) and at 0.5, 1, 6, 24, 48, 96, 168, 264, 336, 432, 504 and 672 h post-dose after the 1st, and the 4th dosing. The LC-MS/MS method was validated to determine the plasma concentrations of goserelin.

### 2.6 Statistical analysis

Quantitative data such as testosterone levels, body weight, food consumption, hematology, clinical chemistry, organ weights and ratios were presented as mean ± standard deviation. Quantitative data were evaluated using one-way analysis of variance (ANOVA). If the ANOVA was significant (*p* ≤ 0.05), Dunnett’s t-test was then used for pairwise comparisons. Levene’s test was used to analyze the variance homogeneity. In the case of heterogeneity of variances, Kruskal–Wallis (K-W) H tests were used to analyze and if there was significant difference (*p* ≤ 0.05), Mann-Whitney (M-W) U tests were used for pairwise comparison. PRISTIMA 6.1.1 system (Xybion Medical Systems Corporation, United States) was used for the statistical analysis of body weight, food consumption, hematology, clinical chemistry, organ weights and ratios. The Graphpad prism 5.0 was used for statistical analysis of the testosterone levels.

## 3 Results

### 3.1 Pharmacology studies

#### 3.1.1 Single-dosing pharmacology study in rats

**FIGURE 1:**
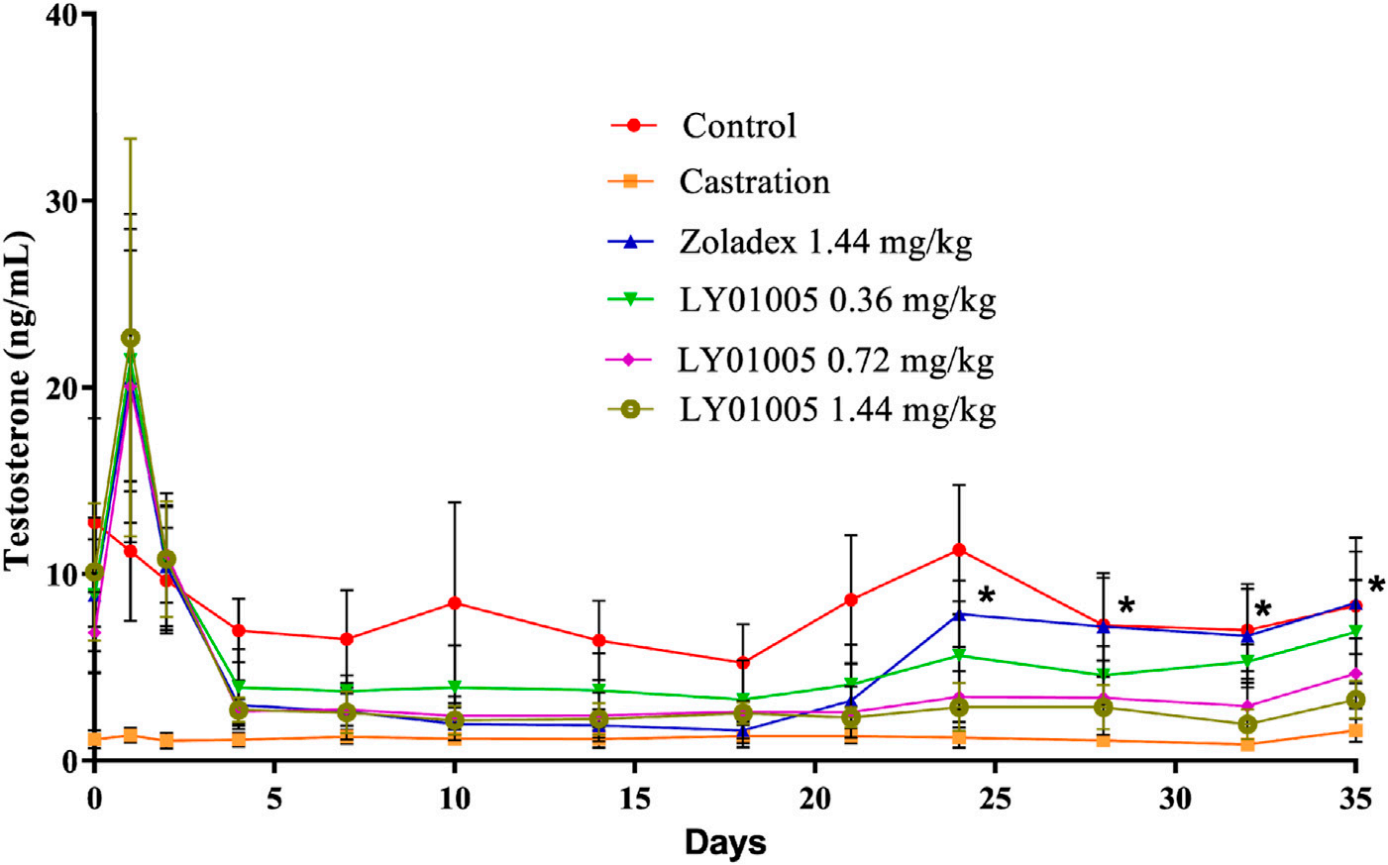
Effects of LY01005 on serum testosterone levels in male rats. *, *p < 0.05*, LY01005 compared to Zoladex^®^ at the same dose.

The pharmacological effects of goserelin mostly result from its occupation of the majority of GnRH receptors present in the pituitary gland. Initially there is a supra-physiological elevation of testosterone and estradiol levels post-dosing in males and in females, respectively. Thereafter, testes atrophy and a decrease in testosterone secretion are observed in males, and ovarian atrophy and a decrease in estradiol to castrate or post-menopausal levels are observed in females due to the long-lasting GnRH receptor blockade. Based on this unique pharmacological mechanism of action of goserelin, here serum testosterone level was chosen as a pharmacodynamics biomarker in male rats. The results showed that the initial supra-physiological elevation of testosterone was as expected at 24 h post-dosing in LY01005-treated rats. Then the testosterone level rapidly fell to castration level on Day 4 and maintained until Day 35 in 0.72 and 1.44 mg/kg LY01005-treated groups. There was no difference between LY01005 and Zoladex^®^ treatments in their effects on the serum testosterone levels from Day 1 to Day 21. However, the serum testosterone level in LY01005-treated rats was significantly lower than that of Zoladex^®^-treated rats from Day 24 to Day 35. The potency of LY01005 was similar to Zoladex^®^ but its effect lasted longer and was more stable (Figure 1).

#### 3.1.2 Multiple-dosing pharmacology study in rats

**FIGURE 2:**
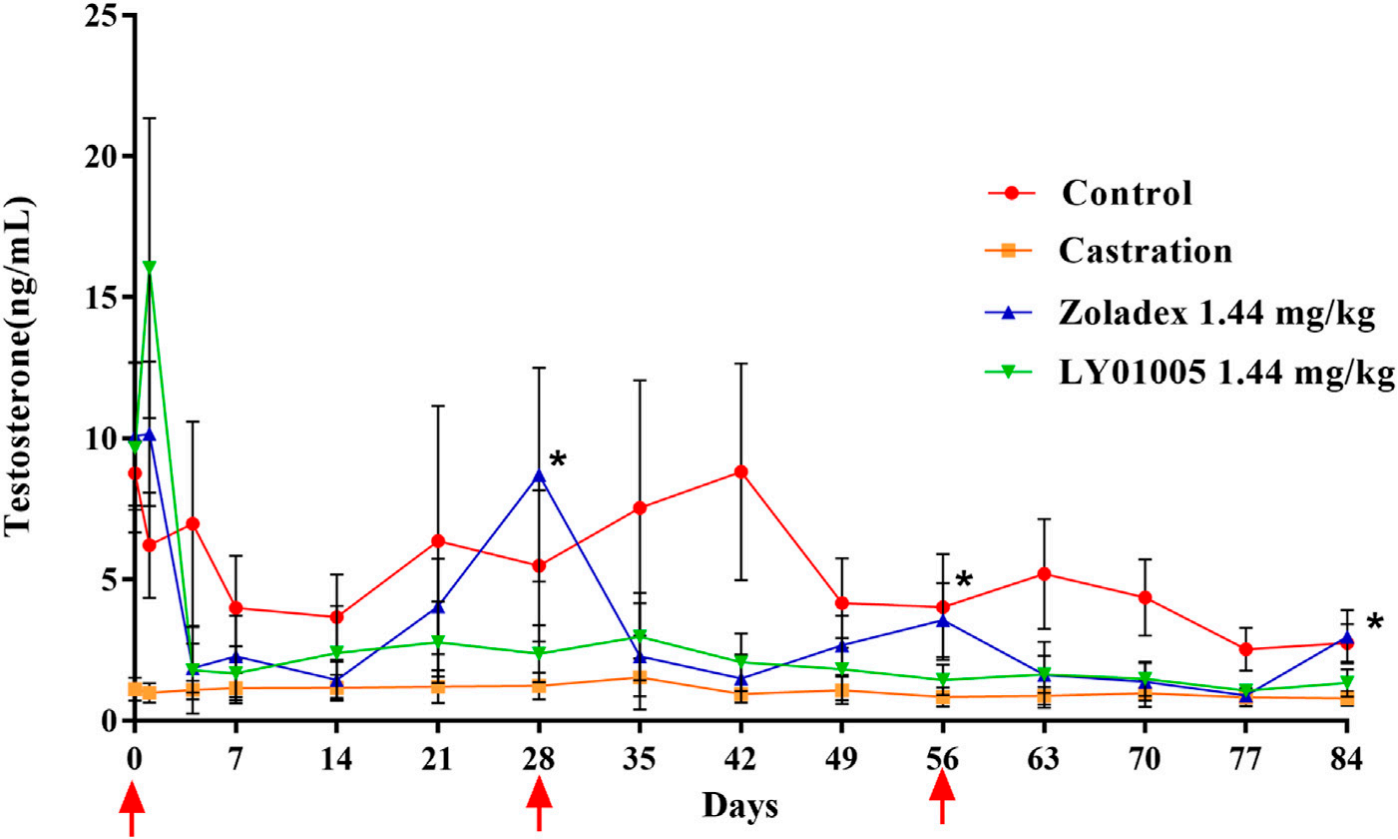
Effects of LY01005 on serum testosterone levels in male rats. *, *p < 0.05*, LY01005 compared to Zoladex^®^ at the same dose.

Acute-on-chronic phenomenon in GnRH agonist therapy refers to the paradoxical increase of serum testosterone level at the end of the dosage interval which is generally attributed to the premature exhaustion of a depot formulation. Therefore, a multiple-dosing pharmacodynamics study is necessary to examine whether the acute-on-chronic phenomenon occurs during LY01005 treatment. As is shown in Figure 2 and as expected, the initial supra-physiological level of testosterone on Day 1 and the rapid reduction to castration level on Day 4 were observed both in LY01005- and Zoladex^®^-treated animals. The testosterone concentrations significantly exceeded the castration level from Day 21 to Day 28 (the second dose), from Day 49 to Day 56 (the third dose), and on Day 84 in Zoladex^®^-treated group. In contrast, the testosterone concentrations maintained at the castration level between Day 4 and Day 84 in LY01005-treated group. These data suggested that LY01005 treatment had a much lower risk than Zoladex^®^ treatment in inducing the acute-on-chronic phenomenon.

#### 3.1.3 Functional observational battery tests in rats

The FOB is a non-invasive procedure designed to better quantify neurotoxic effects in animals resulting from exposure to chemicals in conjunction with other neuropathologic evaluation and/or general toxicity studies. In this study, 1 h after the drug administration, a decrease in walking and/or climbing was observed in rats receiving placebo microspheres or LY01005 10.8 mg/kg treatments. On Days 2, 7, 11 and 28, a reduction in movement grade was observed in rats receiving LY01005 3.6 and 10.8 mg/kg treatment as compared to the vehicle control and the pre-dose data. These changes in movement were most likely due to mechanical stimulation induced by intramuscular injection of placebo microspheres or LY01005 but not due to the pharmacological effect of goserelin. No other abnormalities were observed on the home-cage observation, hand-held observation, open-field observation, stimulus response observation, grip strength and body temperature parameters in the present study. It was concluded that the neurobehavioral functions of the rats were unaffected by the placebo microspheres (287.54 mg/kg) and LY01005 at doses of 1.2, 3.6, 10.8 mg/kg.

#### 3.1.4 Respiratory functions in rats

As shown in Table 1, the parameters of respiration rate, tidal volume and minute ventilation volume in rats receiving placebo microspheres and LY01005 treatments were not significantly different to rats receiving vehicle treatment at 1, 72, 240 and 648 h postdose (*p* > 0.05). The results indicated that the respiratory function of conscious rats was not affected by LY01005 at doses of 1.2, 3.6, 10.8 mg/kg.

**TABLE 1:** Effects of LY01005 on respiratory functions of rats.

Parameters	Time	Vehicle control	Placebo microspheres	LY01005	LY01005	LY01005
				1.2 mg/kg	3.6 mg/kg	10.8 mg/kg
Respiration rate (bpm)	Pre-dose	94.25 ± 20.61	92.70 ± 15.18	93.36 ± 12.17	89.82 ± 16.63	88.41 ± 13.27
	1 h	97.2 ± 23.84	93.65 ± 16.64	91.98 ± 12.37	103.36 ± 41.57	83.66 ± 9.57
	72 h	105.98 ± 24.64	89.83 ± 14.13	102.59 ± 19.38	98.75 ± 17.23	96.06 ± 13.88
	240 h	131.09 ± 60.70	105.72 ± 25.58	100.65 ± 16.14	104.87 ± 12.36	90.75 ± 9.47
	648 h	124.13 ± 89.31	102.43 ± 39.60	96.86 ± 36.03	112.06 ± 30.73	115.09 ± 70.29
Tidal volume (mL)	Pre-dose	1.63 ± 0.69	1.59 ± 0.71	1.38 ± 0.72	1.78 ± 0.86	1.59 ± 0.75
	1 h	1.78 ± 0.88	1.55 ± 0.70	1.40 ± 0.60	1.68 ± 0.79	1.71 ± 0.85
	72 h	1.64 ± 0.61	1.71 ± 0.91	1.75 ± 0.86	1.71 ± 0.54	1.78 ± 0.58
	240 h	2.01 ± 1.11	2.12 ± 1.29	2.11 ± 1.07	1.93 ± 0.83	2.28 ± 1.18
	648 h	1.94 ± 1.22	1.85 ± 1.13	2.29 ± 1.01	2.03 ± 1.44	1.53 ± 0.66
Minute ventilation volume (mL/min)	Pre-dose	143.27 ± 46.35	147.60 ± 69.55	129.54 ± 71.31	160.18 ± 77.50	138.74 ± 65.20
	1 h	158.60 ± 62.23	145.65 ± 74.59	127.71 ± 53.81	167.75 ± 78.14	140.35 ± 66.56
	72 h	171.21 ± 64.54	154.92 ± 90.30	180.53 ± 96.63	166.02 ± 50.20	168.87 ± 57.26
	240 h	238.68 ± 96.18	208.71 ± 102.00	211.10 ± 118.80	200.72 ± 77.95	201.23 ± 90.13
	648 h	184.45 ± 66.87	169.74 ± 64.38	214.60 ± 99.69	210.20 ± 140.78	170.27 ± 108.21

### 3.2 Single-dosing pharmacokinetic study in rats

**FIGURE 3:**
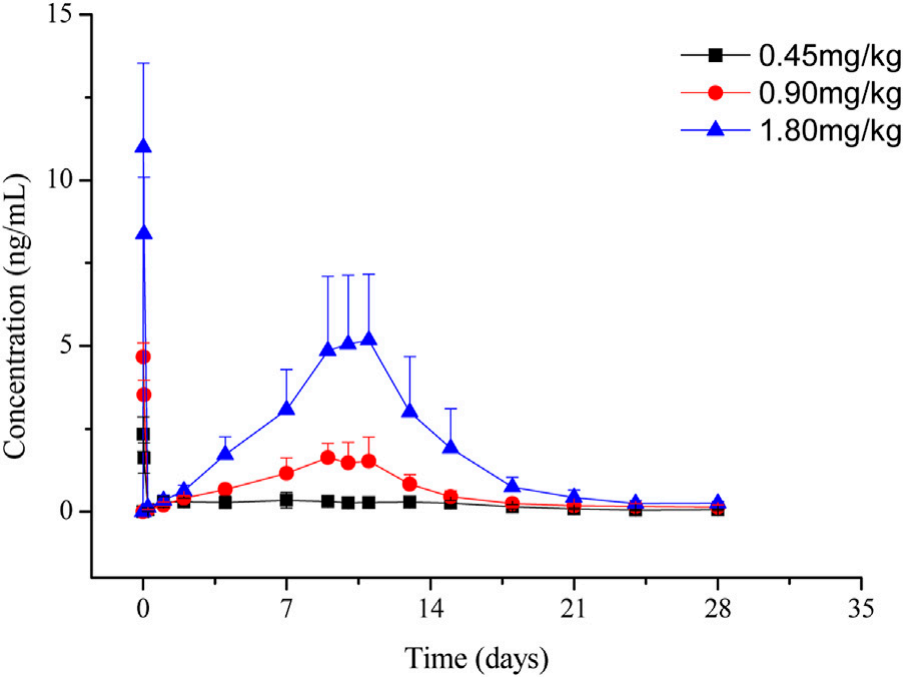
Mean plasma concentration-time profiles of LY01005 after intramuscular injections of 0.45, 0.90 and 1.80 mg/kg (peptide base) in rats.

**FIGURE 4:**
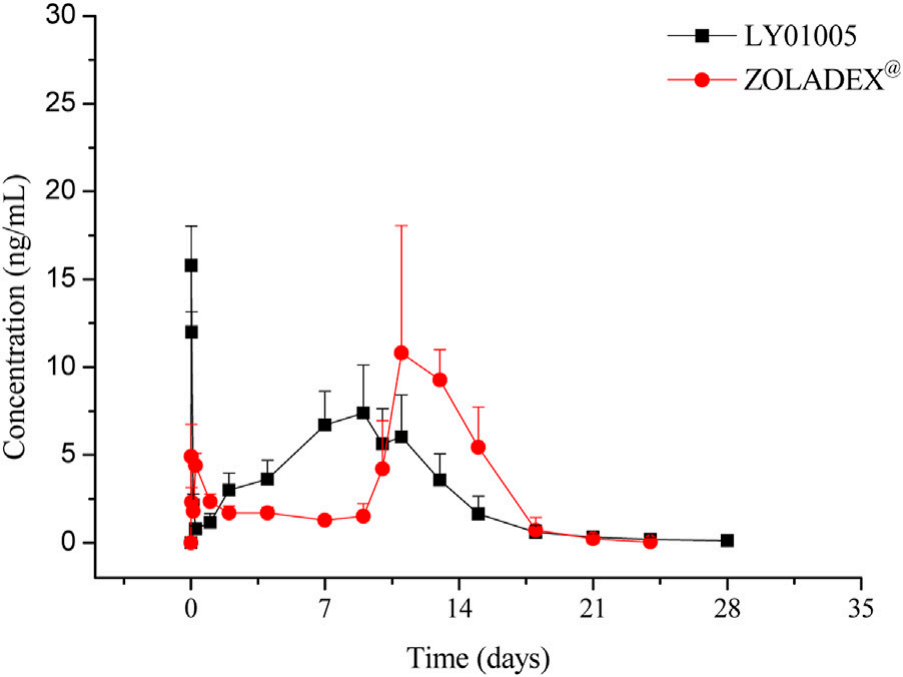
Mean plasma concentration-time profiles of LY01005 and Zoladex^®^ in rats.

The LC-MS/MS method was developed and validated to determine the plasma concentrations of goserelin in rats, with the linearity range of 0.0200 ng/mL (lower limit of quantitation, LLOQ) to 30.0 ng/mL (upper limit of quantification, ULOQ). Following the administration of LY01005, an initial goserelin release was observed at 0.5 h and then a quick decrease within 6 h was observed in the plasma concentration-time profile. Goserelin was continuously released and then a secondary peak was observed from day 5 to day 10. The plasma concentration of goserelin was detectable up to 28 days postdose (Figure 3). As is shown in Table 2, C_max_ and AUC_last_ increased in a dose-proportional manner in the range of 0.45–1.80 mg/kg. The coefficients were 0.962 and 0.983, respectively. The comparison study between LY01005 and Zoladex^®^ at the dosage of 1.80 mg/kg in rats revealed that the plasma concentration of LY01005 was detectable up to 28 days postdose, while Zoladex^®^ was detectable up to 24 days (Table 3; Figure 4). The mean pharmacokinetic parameters of the two groups were shown in Table 2. Compared to Zoladex^®^, the relative bioavailability of LY01005 was 101.0%.

**TABLE 2:** Mean pharmacokinetic parameters of LY01005 in rats.

Dosage		Half-life	T_max1_	T_max2_	C_max1_	C_max2_	AUC_last_
mg/kg		hour	hour	day	ng/mL	ng/mL	hour*ng/mL
0.45	Mean	138.5	0.50	5.17	2.30	0.53	141.2
	SD	57.6	0.00	4.36	0.50	0.12	17.6
0.9	Mean	151.8	0.50	9.5	4.67	1.8	407.6
	SD	76.3	0.00	1.52	0.42	0.45	54.0
1.8	Mean	86.1	0.50	10.2	11.0	6.28	1,206.6
	SD	12.9	0.00	0.98	2.50	1.97	293.8

Note: SD, standard deviation.

**TABLE 3:** Mean pharmacokinetic parameters of LY01005 and Zoladex^®^ in rats.

Drug		Half-life	T_max1_	T_max2_	C_max1_	C_max2_	AUC_last_
		hour	hour	hour	ng/mL	ng/mL	hour*ng/mL
LY01005	Mean	93.0	0.50	196.8	15.8	8.04	1763
	SD	27.4	0.00	26.3	2.2	2.57	352.5
Zoladex^®^	Mean	31.2	0.50	296	4.91	13.0	1746
	SD	5.59	0.00	39.2	1.83	4.51	306

Note: SD, standard deviation.

### 3.3 Toxicology studies

#### 3.3.1 Single-dose toxicity study in rats

In the single-dose toxicity study, rats were i.m. injected with vehicle control (1% SCMC), placebo microspheres, or LY01005 at doses of 3.75, 15 and 60 mg/kg, respectively. All animals survived until the study termination. A decrease in the body weight in male rats was observed in the LY01005-treated groups while an increase in both the body weight and food consumption in female rats were noted in the LY01005-treated groups. Slight decreases in red blood cell (RBC), hemoglobin (HGB) and hematocrit (HCT) and an increase in total bilirubin (TP) were noted in LY01005-treated male rats. In addition, grey-white nodules at the injection site muscles were macroscopically observed in rats receiving placebo microspheres and all doses of LY01005, which were microscopically identified as foreign body granuloma. The relationship of the above changes to LY01005 treatment could not be excluded, which suggests the need of further monitoring in repeated-dose toxicity study. For this acute toxicity study in rats, the maximum tolerated dose (MTD) of LY01005 was determined to be greater than 60 mg/kg.

#### 3.3.2 Sixteen-week subchronic toxicity study in rats

In the subchronic toxicity study, rats were i.m. injected with vehicle (1% SCMC), placebo microspheres (287.54 mg/kg) or LY01005 at 1.2, 3.6 or 10.8 mg/kg, once every 4 weeks for 16 weeks followed by an 8-week recovery period. The signs of swollen and/or scleroma were observed in rats receiving placebo microspheres, 3.6 and 10.8 mg/kg LY01005. During the treatment period, increases in the body weight and food consumption were observed in female rats that received different doses of LY01005. These changes gradually returned back to normal level during the recovery period.

At the end of the treatment period, an increase in lymphocyte % (within 10%) and a decrease in neutrophile granulocyte % (approximately 30%) in all LY01005-treated male rats and an increase in white blood cell and lymphocyte (approximately 50%–60%) were observed in LY01005-treated female rats. These changes were considered to be related to the phagocytosis and degradation of microspheres and chronic inflammation caused by LY01005 injections. Slight decreases in RBC, HGB and HCT were observed in all LY01005-treated male rats. These findings were considered to be related to LY01005-induced hormone changes. Decreases in ALB, TP and A/G were noted in LY01005-treated female rats, which might be related to liver abnormality induced by hormone changes. However, no noticeable change was found in the liver tissues by gross and histopathological examinations or other liver function parameters. As food consumption decrease was observed in female rats, the decrease in ALB and TP might be caused by reduced food intake (Moriyama et al., 2008). The decrease in A/G was caused by ALB decrease. Decreased Ca^2+^ and increased ALP were noted in female rats which were consistent with minimal to slight decrease in bone trabecular number. The findings were considered to be related to LY01005-induced hormone changes. All the changes discussed above were completely recovered to normal at the end of the recovery period.

During the treatment period, significantly increased FSH and LH, progestin and testosterone levels in the early phase, and a decrease or a trend of decrease in the late phase were observed in LY01005-treated rats. These observed hormone changes were directly related to the pharmacological mechanism of LY01005. The changes in hormone levels were completely or partially recovered at the end of the recovery period.

At the completion of treatment, decreases in the absolute weight and organ/body (and brain) weight ratios of the testes, epididymides and prostate were noted in male rats at all LY01005 treatment doses in a dose-dependent manner. Decreases in absolute weight and organ/body (and brain) weight ratios of the ovaries and uterus were observed in female rats at all LY01005 treatment doses in a dose-dependent manner. The changes in the reproductive system (germ cell depletion accompanied by mineralization, sperm number and cellular debris in the epididymal duct, prostate atrophy, and seminal vesicle atrophy in males; ovarian atrophy, uterus atrophy, cervix atrophy, vagina atrophy and mammary glands acinar atrophy in females) were directly related to the pharmacological mechanism of LY01005. These changes in the reproductive system mostly returned back to normal by the end of the recovery period. Moreover, at the end of the recovery period, increased number of follicles in the ovaries and hyperplasia of luminal epithelium/glandular epithelium in the uterus and the vagina epithelium were observed in LY01005-treated female rats; epithelium hyperkeratosis of the cervix were noted in rats receiving 3.6 mg/kg LY01005; epithelium mucification of cervix and vagina were noted in rats receiving 10.8 mg/kg LY01005. These changes could be attributable to LY01005 withdrawal-induced hormone changes.

During the treatment period, slightly decreased bone trabecular, bone marrow hematopoietic cells, and slightly increased bone marrow adipocytes were observed in LY01005-treated rats. These changes were considered secondary effects to LY01005-induced hormone changes.

Local nodules were observed at injection sites in placebo microspheres and all LY01005 treatment groups. Histopathological examination revealed foreign body granuloma in the peripheral connective tissue surrounding sciatic nerve at injection sites. These changes mostly returned back to normal by the end of the recovery period.

**FIGURE 5:**
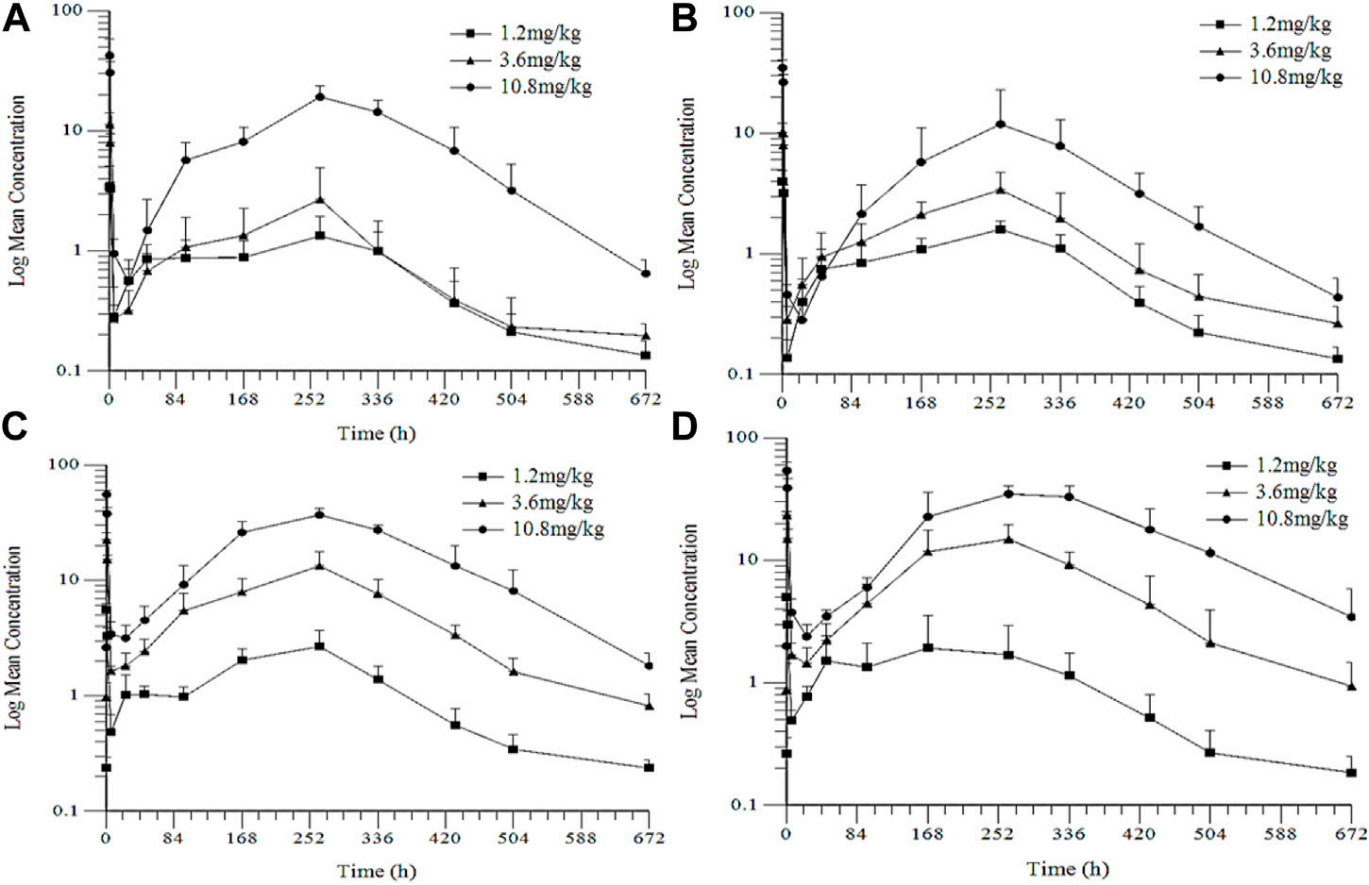
Mean Plasma Concentration-Time Profiles of Goserelin in TK Study in Rats **(A)** and **(C)**: the 1st and the 4th dose in female rats **(B)** and **(D)**: the 1st and 4th dose in male rats.

The LC-MS/MS method was validated to determine the plasma concentrations of goserelin in the toxicokinetics (TK) studies. The linearity range was 0.05–40 ng/mL as presented in Figure 5; Table 4. No significant sex differences were noted in drug exposure (AUC_last_) at each dose after the 1st and 4th dosing. After the 1st dosing, the average drug exposure (AUC_last_) increment was lower than dose-proportionality within 1.2–3.6 mg/kg in female rats, while the AUC_last_ increment was higher than dose-proportionality within 3.6–10.8 mg/kg in female rats. After the 4th dosing, the average drug exposure (AUC_last_) was increased dose-proportionally within 1.2–10.8 mg/kg in female and male rats. The drug accumulation was not apparent in 1.2 mg/kg group (AUC_last_ ratio: 1.51) while the accumulation was apparent in 3.6 (AUC_last_ ratios: 4.96) and 10.8 mg/kg groups (AUC_last_ ratios: 2.81).

**TABLE 4:** Mean TK parameters in rats after intramuscular injection of LY01005.

Day	Dose (mg/kg)	AUClast (h*ng/mL)			Cmax1 (ng/mL)		Cmax2 (ng/mL)		T_max1_(h)	T_max2_(h)
		Mean		SD	Mean	SD	Mean	SD		
	1.2	467.87		127.53	3.98	0.77	1.58	0.24	0.50	264.00
1	3.6	772.36		384.84	11.03	2.07	3.31	1.64	0.50	264.00
	10.8	3,971.86		1984.45	38.80	12.06	15.60	8.84	0.50	264.00
85	1.2	706.46		248.57	5.24	0.64	2.63	1.08	0.50	216.00
	3.6	3,829.30		588.12	22.92	2.75	15.46	4.53	0.50	264.00
	10.8	11,147.11		981.10	54.91	6.98	37.86	5.28	0.50	264.00

Note: T_max1_, T_max2_ were the medians.

In summary, the changes in hormones (FSH, LH, testosterone, progestin) and in reproductive system (uterus, ovary, vagina, cervix uteri, mammary gland, testis, epididymis and prostate) were related to the pharmacological effects of goserelin. The changes in bone and bone marrow were secondary to hormone changes induced by LY01005. In addition, foreign body granulomas at injection sites may be caused by excipient. At the end of the recovery period, these changes returned back to normal.

## 4 Discussion and conclusion

Maintaining effective and sufficient suppression of serum testosterone levels (below 50 ng/dL) is one of the essential strategies in the treatment of metastatic prostate cancer. Currently this is primarily achieved by front-line androgen-deprivation therapy agents such as long-acting GnRH agonists (goserelin, histrelin, leuprolide, and triptorelin) (Scher et al., 2008; Desai et al., 2021). GnRH agonists are known to cause a mechanism-related transient surge in testosterone level and then decrease to the castration level. However, certain patients may fail to reach this primary therapeutic endpoint and may experience significant testosterone fluctuation during long-term maintenance treatment when repeated dosing is required. This clinical phenomenon is known as the end-of-dose phenomenon or acute-on-chronic phenomenon (Sharifi et al., 2002; Yri et al., 2006; Gomella, 2009). The acute-on-chronic phenomenon occurs in approximately 4%–10% of patients who receive GnRH agonists treatment (Berges, 2005). It is an obvious risk for those prostate cancer patients who experience testosterone fluctuation during maintenance treatment to recur (Attard et al., 2009; Eckstein and Haas, 2014).

In the present study, there were no significant differences on the rat serum testosterone levels between LY01005- and Zoladex^®^-treated rats in the first three weeks of dosing. However, LY01005 could be more effective than Zoladex^®^ in the subsequent 2 weeks. Similar findings were also observed in the three-dose pharmacology study in rats. Testosterone concentration was maintained at castration level until the end of the 12-week study period in LY01005-treated rats. In contrast, the testosterone concentration significantly exceeded castration level at the end of each dosing interval in Zoladex^®^-treated rats. Pharmacokinetic results provided a reasonable explanation for the observed pharmacodynamic differences between LY01005 and Zoladex^®^. The plasma concentration of LY01005 was detectable for up to 28 days while Zoladex^®^ was only detectable for 24 days. These data support the notion that LY01005 has less risk of acute-on-chronic phenomenon for clinical prostate cancer treatment as compared to Zoladex^®^.

Safety pharmacology results showed that LY01005 did not show noticeable activity in the central nervous system and respiratory system in rats up to the dose of 10.8 mg/kg, and no noticeable activity in the cardiovascular system in dogs up to the dose of 3.6 mg/kg (unpublished data). Acute and subchronic toxicity studies of LY01005 were conducted in rats and dogs (unpublished data) by intramuscular route of administration. Almost all of the positive findings from these studies including the changes in hormones (FSH, LH, testosterone, progestin) and in reproductive system (uterus, ovary, vagina, cervix uteri, mammary gland, testis, epididymis and prostate) were related to the direct pharmacological effects of goserelin. In addition, slightly decreased bone trabecular, bone marrow hematopoietic cells, and slightly increased bone marrow adipocytes were only observed in LY01005-treated rats. These changes were considered secondary to goserelin (LY01005)-induced hormone changes. Histopathological changes in foreign body removal reaction induced by excipient were also observed. We compared the toxicity-related measures of LY01005 (as reported here) with the published non-clinical safety data of Zoladex^®^ (Goserelin Depot, 2012). No noticeable differences were observed between the two drugs.

In conclusion, LY01005 is an investigational new drug product of goserelin acetate formulated as extended-release microspheres. LY01005 could be administered through a much finer needle to minimize patient’s discomfort and reduce the risk of injection site trauma as compared to Zoladex^®^. LY01005 had a comparable pharmacodynamic potency to Zoladex^®^ in rats but with a more lasting and stable effect. This may be more beneficial in reducing the risk of acute-on-chronic phenomenon when compared to Zoladex^®^. Almost all of the findings reported in toxicity studies were related to the known pharmacological effects and secondary effects to goserelin-induced hormone changes. These data strongly support the proposed clinical investigational plan and marketing application for LY01005.

## Data Availability

The original contributions presented in the study are included in the article/Supplementary Material, further inquiries can be directed to the corresponding authors.
