# Supplementary material for: Pharmacological and toxicological studies of a novel goserelin acetate extended-release microspheres in rats
Source: Front Pharmacol. 2023 Feb 21;14:1125255. doi: 10.3389/fphar.2023.1125255 (PMC9989164; doi:10.3389/fphar.2023.1125255)
Supplement: Supplementary file 1 [file Table1.DOCX]

**Supplementary Table 1**

| **Toxicity studies** | | | | | | | | | | |
| --- | --- | --- | --- | --- | --- | --- | --- | --- | --- | --- |
| **Title：**16-Week Toxicity Study of LY01005 via Intramuscular Injection in Sprague Dawley Rats | | | | | | | **Species/Strain：**Sprague Dawley rats | | | |
| **Duration of Dosing：**Once every 4 weeks for total 4 doses | | | **Dosing Route：**Intramuscular injection | | | | **Recovery Phase：**8-week | | | |
| **Group** | **Vehicle control** | | **Placebo microspheres** | | **LY01005-low dose** | | **LY01005-middle dose** | | **LY01005-high dose** | |
| **Dose level：mg/kg** | **0** | | **287.54** | | **1.2** | | **3.6** | | **10.8** | |
| **Number of animals** | M：15 | F：15 | M：15 | F：15 | M：15 | F：15 | M：15 | F：15 | M：15 | F：15 |
| End of dosing phase | 10 | 10 | 10 | 10 | 10 | 10 | 10 | 10 | 10 | 10 |
| End of recovery phase | 5 | 5 | 5 | 5 | 5 | 5 | 5 | 5 | 5 | 5 |
| **Body Weight（g）** | | | | | | | | | | |
| **Number of animals** | **M：15** | **F：15** | **M：15** | **F：15** | **M：15** | **F：15** | **M：15** | **F：15** | **M：15** | **F：15** |
| Day 8 | 274.9 ±14.9 | 200.9 ± 10.6 | 274.1 ± 16.2 | 204.5 ± 9.1 | 273.3 ± 16.3 | 213.6 ± 13.5^*^ | 266.7 ± 21.2 | 210.8 ± 9.0^*^ | 262.9 ±15.0 | 216.8 ±10.2^*#^ |
| Day 15 | 313.5 ± 19.0 | 222.1 ±14.4 | 312.7 ± 22.3 | 228.2 ± 10.9 | 317.7 ±23.4 | 245.9 ± 15.4^*#^ | 309.2 ± 25.7 | 244.9 ± 9.8^*#^ | 306.3 ± 20.6 | 251.4 ± 13.7^*#^ |
| Day 22 | 344.8 ± 25.3 | 232.7± 16.1 | 343.9 ± 25.9 | 241.9 ± 13.4 | 351.4 ±28.7 | 270.3 ± 18.0^*#^ | 340.6± 32.5 | 269.5 ± 8.1^*#^ | 336.6 ± 24.8 | 277.3 ± 15.4^*#^ |
| Day 29 | 372.9 ± 27.1 | 245.6 ± 16.0 | 375.5 ±30.4 | 252.4 ± 15.1 | 384.7 ± 33.3 | 283.7 ± 19.2^*#^ | 368.4 ±34.7 | 285.2 ± 8.8^*#^ | 363.9 ±27.3 | 292.4 ± 18.2^*#^ |
| Day 36 | 392.6 ± 31.6 | 253.3 ± 16.4 | 397.2 ± 34.9 | 259.6 ± 14.3 | 410.4± 37.0 | 297.5 ± 19.3^*#^ | 396.5 ± 39.2 | 297.7 ± 11.0^*#^ | 387.7 ± 29.7 | 305.9 ± 19.3^*#^ |
| Day 43 | 412.6 ± 33.9 | 257.5 ± 17.6 | 419.0 ± 39.8 | 266.5 ± 16.7 | 430.9 ± 41.3 | 307.1 ± 21.8^*#^ | 413.2± 40.4 | 310.3 ± 13.4^*#^ | 408.9± 33.6 | 319.8 ± 21.2^*#^ |
| Day 50 | 428.2 ± 37.9 | 264.9 ± 19.1 | 438.7± 44.9 | 272.0 ± 17.3 | 449.4 ± 45.5 | 317.9 ± 22.2^*#^ | 432.7 ± 45.0 | 320.0± 15.2^*#^ | 424.1 ± 40.8 | 327.2 ± 22.4^*#^ |
| Day 57 | 440.2± 40.3 | 268.9 ± 18.3 | 452.7 ± 49.2 | 277.3 ± 18.9 | 465.2 ± 50.4 | 325.9 ± 23.2^*#^ | 447.1 ± 48.4 | 328.6 ± 15.4^*#^ | 439.3 ± 40.8 | 334.8 ± 23.9^*#^ |
| Day 64 | 451.8± 41.1 | 273.1 ± 20.3 | 463.4 ± 50.0 | 283.3 ± 20.8 | 479.4 ± 54.8 | 331.6 ± 25.0^*#^ | 457.7 ± 50.0 | 332.7 ± 17.0^*#^ | 451.3 ± 42.5 | 341.4 ± 25.0^*#^ |
| Day 71 | 463.6 ± 42.5 | 278.7 ± 19.1 | 478.3 ± 51.5 | 287.7 ± 21.4 | 486.1 ± 55.3 | 336.6 ± 23.4^*#^ | 469.1 ± 50.9 | 338.8 ± 18.3^*#^ | 459.2 ± 41.1 | 346.8 ± 25.6^*#^ |
| Day 78 | 475.1 ± 46.4 | 279.0 ± 20.6 | 489.5± 53.8 | 291.3± 22.1 | 497.1 ± 57.7 | 339.8 ± 24.5^*#^ | 480.8 ± 54.1 | 344.2 ± 18.3^*#^ | 466.6 ± 44.2 | 351.9 ± 26.3^*#^ |
| Day 85 | 489.7 ± 49.7 | 285.1 ± 19.1 | 502.9 ± 56.7 | 295.9 ± 24.6 | 509.5 ± 58.9 | 345.7 ±24.4^*#^ | 493.0 ± 56.4 | 349.7 ± 20.6^*#^ | 480.7± 47.5 | 356.3 ± 28.5^*#^ |
| Day 92 | 497.3 ± 51.3 | 285.8 ± 18.1 | 507.5 ± 58.8 | 298.7 ± 22.1 | 519.6± 61.5 | 347.6 ± 25.2^*#^ | 500.8± 57.5 | 352.1 ± 21.6^*#^ | 485.7± 45.6 | 359.0± 29.2^*#^ |
| Day 99 | 504.0 ± 52.0 | 289.9 ± 20.3 | 511.5 ± 59.3 | 303.8± 24.1 | 521.8± 64.7 | 352.1 ± 26.5^*#^ | 503.7 ± 58.0 | 355.1 ± 22.4^*#^ | 490.7 ± 46.9 | 363.4 ± 31.2^*#^ |
| Day 106 | 509.0 ± 53.3 | 291.1 ± 22.2 | 520.4 ± 59.3 | 304.5 ± 24.2 | 528.8 ± 64.5 | 358.6 ± 26.3^*#^ | 514.9 ± 61.3 | 360.7 ± 23.6^*#^ | 500.2 ± 47.9 | 368.4 ± 31.6^*#^ |
| Day 113 | 486.3± 50.9 | 318.5 ± 22.9 | 491.4 ± 64.0 | 319.6 ± 32.6 | 526.9 ± 54.6 | 356.9 ± 33.6 | 471.1± 48.2 | 374.8 ± 34.4^*^ | 506.9 ± 47.8 | 350.6 ± 28.5 |
| **Number of animals** | **M：5** | **F：5** | **M：5** | **F：5** | **M：5** | **F：5** | **M：5** | **F：5** | **M：5** | **F：4** |
| Recovery day 8 | 493.2 ± 43.7 | 310.7 ± 20.9 | 494.1± 61.7 | 320.5 ± 36.9 | 528.9 ± 56.0 | 358.3 ± 33.5 | 474.8 ± 48.3 | 373.9± 32.5^*^ | 504.6 ± 39.6 | 352.8 ± 29.2 |
| Recovery day 15 | 499.9 ± 45.3 | 308.8 ± 22.7 | 498.6 ± 68.0 | 324.1 ± 40.9 | 530.3 ± 54.9 | 360.9 ± 35.1 | 476.1± 49.0 | 378.8 ± 35.1^*^ | 508.4 ± 45.8 | 353.9 ± 28.9 |
| **Hormone -** **24 hrs after the 1^st^ dosing** | | | | | | | | | | |
| Progestin(ng/mL) | 0.237± 0.047 | 4.809± 1.549 | 0.211± 0.054 | 3.717± 0.823 | 3.513± 0.404^*#^ | 4.671±1.211 | 4.346± 0.380^*#^ | 4.411± 0.382 | 3.799± 1.408^*#^ | 4.634± 0.384 |
| Luteinizing hormone(ng/mL) | 1.821± 1.204 | 1.560± 0.733 | 0.640± 0.154 | 1.855± 0.429 | 11.967±2.459^*#^ | 6.597± 1.116^*#^ | 9.637± 1.216^*#^ | 6.015± 1.247^*#^ | 7.877± 1.442^*#^ | 7.399± 1.715^*#^ |
| Testosterone(ng/mL) | 2.476± 0.198 | 1.911± 0.752 | 11.787± 6.893 | 2.700± 0.675 | 18.711± 7.849^*^ | 2.459± 0.577 | 23.723± 3.687^*^ | 3.227± 0.748 | 18.395± 8.531^*^ | 6.279± 8.958 |
| **Hormone - 4 days after the 1^st^ dosing** | | | | | | | | | | |
| Progestin(ng/mL) | 0.390± 0.061 | 3.820±0.820 | 0.537± 0.212 | 4.537± 0.309 | 0.448± 0.249 | 3.168± 0.825 | 1.245± 1.068 | 3.798± 0.603 | 2.981± 0.793^*#^ | 3.993± 0.708 |
| Follicle-stimulating hormone (ng/mL) | 10.186± 4.416 | 5.312± 5.004 | 8.233± 1.210 | 5.106± 3.720 | 2.537± 1.105^*#^ | 1.250± 0.344^#^ | 4.540± 1.360^*#^ | 0.667± 0.369^*#^ | 4.887± 1.240^*#^ | 1.267± 0.440^#^ |
| Luteinizing hormone(ng/mL) | 1.104± 0.151 | 2.729± 1.645 | 1.828± 1.397 | 4.112± 3.856 | 1.979± 1.530 | 3.970± 1.127 | 3.576± 0.812^*^ | 3.936± 0.803 | 3.219± 1.293 | 5.112± 1.070 |
| Testosterone(ng/mL) | 4.640± 3.708 | 2.474± 0.050 | 4.217± 2.823 | 2.427± 0.403 | 3.823± 1.031 | 2.536± 0.648 | 3.235± 0.449 | 4.084± 1.229^*#^ | 3.570± 0.993 | 2.721± 0.741 |
| **Hormone - 27 days after the 1^st^ dosing** | | | | | | | | | | |
| Progestin(ng/mL) | 0.392±0.276 | 4.883± 0.936 | 0.291± 0.313 | 6.067± 0.659 | 0.512±0.245 | 2.413± 0.498^*#^ | 0.359±0.320 | 2.688± 1.008^*#^ | 0.707± 0.490 | 2.049± 1.082^*#^ |
| Follicle-stimulating hormone (ng/mL) | 10.142± 2.230 | 6.108± 7.371 | 8.281± 0.773 | 12.833± 4.434 | 3.412± 1.066^*#^ | 1.682± 0.280^#^ | 4.144± 1.447^*#^ | 0.915± 0.476^*#^ | 3.320± 0.947^*#^ | 0.755±0.328^*#^ |
| Testosterone(ng/mL) | 5.117± 4.232 | 3.028± 0.245 | 4.955± 2.046 | 3.273± 0.113 | 2.882±0.547 | 1.518± 0.428^*#^ | 3.252± 0.325 | 1.661± 0.410^*#^ | 3.479± 1.094 | 1.856± 0.225^*#^ |
| **Hormone - 27 days after the 2^nd^ dosing** | | | | | | | | | | |
| Progestin(ng/mL) | 0.452± 0.611 | 4.841± 1.600 | 0.914± 0.511 | 4.853± 0.745 | 0.429± 0.217 | 1.209± 0.524^*#^ | 0.561± 0.385 | 1.497± 0.507^*#^ | 0.450± 0.535 | 1.002± 0.282^*#^ |
| Follicle-stimulating hormone (ng/mL) | 7.306± 1.265 | 4.238± 3.841 | 6.770± 2.218 | 7.304± 3.512 | 2.241± 0.912^*#^ | 1.132± 0.314^#^ | 3.335± 1.751^*#^ | 0.469±0.243^*#^ | 2.558± 1.208^*#^ | 0.335± 0.204^*#^ |
| Luteinizing hormone(ng/mL) | 2.062± 1.548 | 1.798± 1.150 | 2.280± 1.444 | 2.852± 1.141 | 0.576± 0.187^*#^ | 2.679± 0.664 | 0.658± 0.229^#^ | 1.814± 0.556 | 0.603± 0.157^#^ | 1.859± 0.390 |
| Testosterone(ng/mL) | 2.824± 0.514 | 2.777± 0.579 | 3.417± 0.752 | 2.627± 0.415 | 2.672± 0.542 | 1.327± 0.383^*#^ | 3.213± 0.953 | 1.780± 0.642^*#^ | 2.625± 1.339 | 1.499± 0.358^*#^ |
| **Hormone - 27 days after the 4^th^ dosing** | | | | | | | | | | |
| Follicle-stimulating hormone (ng/mL) | 5.582± 1.117 | 2.044± 0.807 | 6.262± 0.915 | 5.410± 5.444 | 1.136± 0.530^*#^ | 0.900± 0.206^*#^ | 1.744± 0.935^*#^ | 0.491± 0.280^*#^ | 2.577± 1.832^*#^ | 0.321± 0.211^*#^ |
| Luteinizing hormone(ng/mL) | 1.798± 0.714 | 1.497± 0.341 | 2.131± 0.371 | 5.026± 3.418^*^ | 0.425± 0.079^*#^ | 2.542± 0.655 | 0.548± 0.137^*#^ | 1.909± 0.500^#^ | 0.519± 0.121^*#^ | 1.673± 0.561^#^ |
| Testosterone(ng/mL) | 2.920± 0.916 | 2.115± 0.499 | 2.713± 0.428 | 2.367± 0.497 | 2.472± 0.182 | 1.498± 0.369^#^ | 3.562± 1.158 | 1.713± 0.438 | 3.257± 1.100 | 2.042± 0.368 |
| **Hormone - end of recovery phase** | | | | | | | | | | |
| Progestin(ng/mL) | 0.691± 0.947 | 1.794± 1.165 | 1.233±1.268 | 3.107± 0.931 | 0.686± 0.450 | 5.066± 1.438^*^ | 1.784± 1.491 | 2.460± 1.400 | 1.614± 1.009 | 3.945± 1.820 |
| Follicle-stimulating hormone (ng/mL) | 6.293± 1.064 | 3.478± 2.346 | 7.255± 2.571 | 3.988± 2.713 | 5.770± 4.402 | 2.731± 1.226 | 4.205± 0.802^*#^ | 2.906± 0.823 | 2.012± 0.727^*#^ | 2.537± 1.410 |
| Luteinizing hormone(ng/mL) | 2.217± 0.735 | 1.694± 0.497 | 2.244± 0.218 | 3.244± 2.120 | 1.168± 0.172^*#^ | 1.333± 0.821 | 0.217±0.163^*#^ | 2.416± 1.181 | 0.547± 0.371^*#^ | 1.092± 0.463^#^ |
| **Organ Weight** *-* **End of dosing phase** | | | | | | | | |  |  |
| **Number of animals** | **M：10** | **F：10** | **M：10** | **F：10** | **M：10** | **F：10** | **M：10** | **F：10** | **M：10** | **F：10** |
| Testis | | | | | | | | | | |
| Absolute organ weight（g） | 3.2810 | - | 3.2093 | - | 2.1750^*#^ | - | 2.0233^*#^ | - | 1.9762^*#^ | - |
| Organ-to-body weight ratio （%） | 0.6488 | - | 0.6217 | - | 0.4125^*#^ | - | 0.3796^*#^ | - | 0.4070^*#^ | - |
| Organ-to-brain weight ratio（%） | 149.56 | - | 149.72 | - | 100.48^*#^ | - | 93.13^*#^ | - | 94.35^*#^ | - |
| Epididymides | | | | | | | | | | |
| Absolute organ weight（g） | 1.2795 | - | 1.3810 | - | 0.9347^*#^ | - | 0.9479^*#^ | - | 0.9986^*#^ | - |
| Organ-to-body weight ratio （%） | 0.2534 | - | 0.2677 | - | 0.1780^*#^ | - | 0.1799^*#^ | - | 0.2038^*#^ | - |
| Organ-to-brain weight ratio（%） | 58.32 | - | 64.38 | - | 43.15^*#^ | - | 43.75^*#^ | - | 47.81^#^ | - |
| Ovary | | | | | | | | | | |
| Absolute organ weight（g） | - | 0.0691 | - | 0.0789 | - | 0.0190^*#^ | - | 0.0130^*#^ | - | 0.0131^*#^ |
| Organ-to-body weight ratio （%） | - | 0.0250 | - | 0.0273 | - | 0.0053^*#^ | - | 0.0037^*#^ | - | 0.0035^*#^ |
| Organ-to-brain weight ratio（%） | - | 3.53 | - | 4.02 | - | 0.93^*#^ | - | 0.67^*#^ | - | 0.65^*#^ |
| Uterus | | | | | | | | | | |
| Absolute organ weight（g） | - | 0.6615 | - | 0.6077 | - | 0.1381^*#^ | - | 0.1362^*#^ | - | 0.1319^*#^ |
| Organ-to-body weight ratio （%） | - | 0.2387 | - | 0.2105 | - | 0.0388^*#^ | - | 0.0389^*#^ | - | 0.0355^*#^ |
| Organ-to-brain weight ratio（%） | - | 34.11 | - | 31.01 | - | 6.78^*#^ | - | 6.96^*#^ | - | 6.54^*#^ |
| Prostate | | | | | | | | | | |
| Absolute organ weight（g） | 1.3816 | - | 1.4556 | - | 0.7135^*#^ | - | 0.6465^*#^ | - | 0.6486^*#^ | - |
| Organ-to-body weight ratio（%） | 0.2769 | - | 0.2831 | - | 0.1376^*#^ | - | 0.1198^*#^ | - | 0.1313^*#^ | - |
| Organ-to-brain weight ratio（%） | 63.20 | - | 67.68 | - | 33.24^*#^ | - | 29.56^*#^ | - | 31.14^*#^ | - |
| **Organ Weight** *-* **End of recovery phase** | | | | | | | | |  |  |
| **Number of animals** | **M：5** | **F：5** | **M：5** | **F：5** | **M：5** | **F：5** | **M：5** | **F：5** | **M：5** | **F：5** |
| Testis | | | | | | | | | | |
| Absolute organ weight（g） | 3.2714 | - | 3.1404 | - | 2.6400^*^ | - | 2.1938^*#^ | - | 2.4734^*#^ | - |
| Organ-to-body weight ratio （%） | 0.6619 | - | 0.6338 | - | 0.4906^*#^ | - | 0.4477^*#^ | - | 0.4767^*#^ | - |
| Organ-to-brain weight ratio（%） | 168.47 | - | 143.73 | - | 124.72^*^ | - | 105.80^*#^ | - | 115.44^*#^ | - |
| Epididymides | | | | | | | | | | |
| Absolute organ weight（g） | 1.4110 | - | 1.3592 | - | 1.1126^*#^ | - | 0.8874^*#^ | - | 1.0746^*#^ | - |
| Organ-to-body weight ratio （%） | 0.2852 | - | 0.2743 | - | 0.2082^*#^ | - | 0.1822^*#^ | - | 0.2066^*#^ | - |
| Organ-to-brain weight ratio（%） | 71.86 | - | 62.20 | - | 52.61^*^ | - | 42.76^*#^ | - | 50.13^*#^ | - |
| Ovary | | | | | | | | | | |
| Absolute organ weight（g） | - | 0.0732 | - | 0.0652 | - | 0.0742 | - | 0.0396^*^ | - | 0.0368^*^ |
| Organ-to-body weight ratio （%） | - | 0.0235 | - | 0.0200 | - | 0.0232 | - | 0.0115^*^ | - | 0.0110^*^ |
| Organ-to-brain weight ratio（%） | - | 3.66 | - | 3.34 | - | 3.70 | - | 2.01^*^ | - | 1.83^*^ |
| Uterus | | | | | | | | | | |
| Absolute organ weight（g） | - | 0.7066 | - | 0.9424 | - | 0.4642^#^ | - | 0.5060^#^ | - | 0.4122^#^ |
| Organ-to-body weight ratio （%） | - | 0.2315 | - | 0.2858 | - | 0.1431^#^ | - | 0.1458^#^ | - | 0.1255^#^ |
| Organ-to-brain weight ratio（%） | - | 35.68 | - | 48.07 | - | 22.95^#^ | - | 25.65^#^ | - | 20.54^#^ |
| Prostate | | | | | | | | | | |
| Absolute organ weight（g） | 1.4796 | - | 1.2304 | - | 1.1704 | - | 0.8190 | - | 0.9620 | - |
| Organ-to-body weight ratio （%） | 0.2976 | - | 0.2532 | - | 0.2203 | - | 0.1697 | - | 0.1848 | - |
| Organ-to-brain weight ratio（%） | 74.93 | - | 56.16 | - | 55.37 | - | 39.51^*^ | - | 44.72^*^ | - |
| **Dosing Phase Necropsy** | | | | | | | | |  |  |
| **Number of animals** | **M：10** | **F：10** | **M：10** | **F：10** | **M：10** | **F：10** | **M：10** | **F：10** | **M：10** | **F：10** |
| **Histological finding-placebo microsphere/LY01005 microscopic related** | | | | | | | | | | |
| Muscles of injection site（dosing sites） | | | | | | | | | | |
| Foreign body granuloma-minimal | 0 | 0 | 3 | 7 | 1 | 1 | 2 | 7 | 2 | 4 |
| Foreign body granuloma -slight | 0 | 0 | 1 | 2 | 0 | 0 | 1 | 1 | 4 | 5 |
| Foreign body granuloma -moderate | 0 | 0 | 0 | 0 | 0 | 0 | 0 | 1 | 4 | 1 |
| **Histological finding- LY01005 related** | | | | | | | | | | |
| Testis | | | | | | | | | | |
| Atrophy, seminiferous tubule -minimal | 0 | - | 0 | - | 5 | - | 5 | - | 4 | - |
| Atrophy, seminiferous tubule-slight | 0 | - | 0 | - | 0 | - | 2 | - | 4 | - |
| Atrophy, seminiferous tubule-moderate | 0 | - | 0 | - | 0 | - | 3 | - | 0 | - |
| Atrophy, seminiferous tubule-marked | 0 | - | 0 | - | 0 | - | 0 | - | 1 | - |
| Atrophy, seminiferous tubule-severe | 1 | - | 0 | - | 1 | - | 0 | - | 0 | - |
| Epididymides | | | | | | | | | | |
| Decreased number, sperm，luminal-minimal | 0 | - | 0 | - | 1 | - | 0 | - | 0 | - |
| Decreased number, sperm，lumina-slight | 0 | - | 0 | - | 0 | - | 1 | - | 1 | - |
| Decreased number, sperm，lumina-marked | 0 | - | 0 | - | 1 | - | 0 | - | 0 | - |
| Decreased number, sperm，lumina-severe | 1 | - | 0 | - | 1 | - | 1 | - | 0 | - |
| Prostate | | | | | | | | | | |
| Atrophy-minimal | 0 | - | 0 | - | 4 | - | 7 | - | 5 | - |
| Atrophy-slight | 0 | - | 0 | - | 4 | - | 1 | - | 1 | - |
| Seminal vesicles | | | | | | | | | | |
| Atrophy-minimal | 0 | - | 0 | - | 4 | - | 2 | - | 4 | - |
| Atrophy-slight | 0 | - | 0 | - | 3 | - | 3 | - | 3 | - |
| Atrophy-moderate | 0 | - | 0 | - | 1 | - | 1 | - | 0 | - |
| Overies | | | | | | | | | | |
| Decreased number, corpus luteum -slight | - | 0 | - | 0 | - | 2 | - | 0 | - | 0 |
| Decreased number, corpus luteum -moderate | - | 0 | - | 0 | - | 1 | - | 3 | - | 0 |
| Decreased number, corpus luteum -marked | - | 0 | - | 0 | - | 2 | - | 2 | - | 1 |
| Decreased number, corpus luteum -severe | - | 0 | - | 0 | - | 4 | - | 4 | - | 9 |
| Decreased number, follicles -marked | - | 0 | - | 0 | - | 3 | - | 2 | - | 5 |
| Decreased number, follicles -severe | - | 0 | - | 0 | - | 7 | - | 8 | - | 5 |
| Uterus | | | | | | | | | | |
| Atrophy-marked | - | 0 | - | 0 | - | 9 | - | 5 | - | 5 |
| Atrophy-severe | - | 0 | - | 0 | - | 1 | - | 5 | - | 5 |
| Cervix | | | | | | | | | | |
| Atrophy-minimal | - | 0 | - | 0 | - | 0 | - | 1 | - | 0 |
| Atrophy-slight | - | 0 | - | 0 | - | 2 | - | 0 | - | 0 |
| Atrophy-severe | - | 0 | - | 0 | - | 8 | - | 9 | - | 10 |
| Vagina | | | | | | | | | | |
| Atrophy-minimal | - | 0 | - | 0 | - | 3 | - | 2 | - | 0 |
| Atrophy-slight | - | 0 | - | 0 | - | 3 | - | 3 | - | 2 |
| Atrophy-moderate | - | 0 | - | 0 | - | 4 | - | 5 | - | 8 |
| **Recovery Phase Necropsy** | | | | | | | | | | |
| **Number of animals** | **M：5** | **F：5** | **M：5** | **F：5** | **M：5** | **F：5** | **M：5** | **F：5** | **M：5** | **F：5** |
| **Histological finding- LY01005 related** | | | | | | | | | | |
| Testes | | | | | | | | | | |
| Atrophy, seminiferous tubule -minimal | 0 | - | 0 | - | 1 | - | 3 | - | 2 | - |
| Atrophy, seminiferous tubule -slight | 0 | - | 0 | - | 1 | - | 0 | - | 2 | - |
| Atrophy, seminiferous tubule -moderate | 0 | - | 0 | - | 0 | - | 1 | - | 0 | - |
| Mineralization-minimal | 0 | - | 0 | - | 2 | - | 1 | - | 1 | - |
| Mineralization -slight | 0 | - | 0 | - | 0 | - | 1 | - | 1 | - |
| Epididymides | | | | | | | | | | |
| Decreased mumber, sperm, luminal-minimal | 0 | - | 0 | - | 0 | - | 0 | - | 1 | - |
| Protate | | | | | | | | | | |
| Atrophy-minimal | 0 | - | 0 | - | 0 | - | 4 | - | 3 | - |
| Atrophy-slight | 0 | - | 0 | - | 0 | - | 0 | - | 1 | - |
| Seminal vesicles | | | | | | | | | | |
| Atrophy-minimal | 0 | - | 0 | - | 2 | - | 3 | - | 2 | - |
| Overies | | | | | | | | | | |
| Decreased number, corpus luteum -slight | - | 0 | - | 0 | - | 0 | - | 0 | - | 1 |
| Decreased number, corpus luteum -moderate | - | 0 | - | 0 | - | 2 | - | 0 | - | 0 |
| Decreased number, corpus luteum -marked | - | 0 | - | 0 | - | 0 | - | 1 | - | 2 |
| Decreased number, corpus luteum -severe | - | 0 | - | 0 | - | 1 | - | 3 | - | 2 |
| Increased number, follicles -minimal | - | 0 | - | 0 | - | 1 | - | 2 | - | 1 |
| Increased number, follicles -slight | - | 0 | - | 0 | - | 1 | - | 1 | - | 2 |
| Increased number, follicles -moderate | - | 0 | - | 0 | - | 0 | - | 0 | - | 1 |
| Decreased number, follicles -moderate | - | 0 | - | 0 | - | 0 | - | 0 | - | 1 |
| Uterus | | | | | | | | | | |
| Atrophy-minimal | - | 0 | - | 0 | - | 4 | - | 2 | - | 4 |
| Atrophy-slight | - | 0 | - | 0 | - | 1 | - | 3 | - | 1 |
| Hyperplasia，luminal epithelial/glandular epithelial -minimal | - | 0 | - | 0 | - | 4 | - | 2 | - | 3 |
| Hyperplasia，luminal epithelial/glandular epithelial -slight | - | 0 | - | 0 | - | 1 | - | 2 | - | 2 |
| Cervix | | | | | | | | | | |
| Atrophy-minimal | - | 0 | - | 0 | - | 1 | - | 1 | - | 1 |
| Atrophy-slight | - | 0 | - | 0 | - | 1 | - | 0 | - | 0 |
| Hyperplasia，luminal epithelial/glandular epithelial -minimal | - | 0 | - | 0 | - | 0 | - | 2 | - | 0 |
| Hyperplasia，luminal epithelial/glandular epithelial -slight | - | 0 | - | 0 | - | 0 | - | 0 | - | 1 |
| Hyperkeratosis，epithelial -minima | - | 0 | - | 0 | - | 0 | - | 1 | - | 0 |
| Vagina | | | | | | | | | | |
| Atrophy-minimal | - | 0 | - | 0 | - | 2 | - | 0 | - | 0 |
| Atrophy-slight | - | 0 | - | 0 | - | 0 | - | 0 | - | 2 |
| Hyperplasia，epithelial -minimal | - | 0 | - | 0 | - | 1 | - | 1 | - | 2 |
| Mucilaginous，epithelial -minimal | - | 0 | - | 0 | - | 0 | - | 0 | - | 2 |

^*^ Statistical significance was found when compared with the vehicle control group (P≤0.05)；^#^Statistical significance was found when compared with the placebo microspheres group (P≤0.05). “ - ”= Not Applicable
